# Complete genome sequence of *Gibbsiella dentisursi* strain 2e isolated from the gut of *Apriona swainsoni*

**DOI:** 10.1128/mra.00390-26

**Published:** 2026-08-04

**Authors:** Lei Zhang, Changlong Xie, Xiaoran Han, Huijun Li, Daqiang Wu

**Affiliations:** 1 Department of Resources and Environment, Anhui Vocational and Technical College of Forestry, Hefei, China; 2 Department of Pathogenic Biology and Immunology, College of Integrated Chinese and Western Medicine, Anhui University of Chinese Medicine 117843, Hefei, China; Portland State University, Portland, Oregon, USA

**Keywords:** *Gibbsiella dentisursi*, gut microbiota, *Apriona swainsoni*

## Abstract

*Gibbsiella dentisursi* strain 2e was isolated from the gut of *Apriona swainsoni* larvae collected in China. Here, we present the complete genome sequence of this strain to investigate its possible function in the lignocellulolytic catabolism and its functional roles in nitrogen utilization, including nitrogen fixation and urea reduction.

## ANNOUNCEMENT

*Gibbsiella dentisursi* strain 2e was isolated from the posterior hindgut of *Apriona swainsoni* larvae collected on 30 October 2023, in Zhejiang, China (latitude 29°03′53.64″ N, longitude 121°16′059.69″ E), anaerobically on Ashby nitrogen-free agar plates (1). Here, we sequenced the genome of strain 2e to investigate its possible role in degrading lignocellulose and fixing atmospheric nitrogen (1–4). The taxonomic identification of strain 2e was determined by 27F-1492R 16S rRNA gene sequence alignment to GenBank with the best hit of *G. dentisursi*.

Genomic DNA was extracted with the sodium chloride-tris-EDTA (STE) method (5) from bacteria cultured at 37°C in lysogeny broth (LB) liquid medium (6) with shaking at 180 rpm under aerobic conditions. In Oxford Nanopore Technologies (ONT) library preparation, first, 15 kb large fragments of DNA, which were fragmented from gDNA by the Covaris S220 system, were recovered by the Blue Pippin recovery system and then repaired (7). A barcode was added by the PCR-free method of the EXP-NBD104 Kit of ONT. The ligation-based protocol was used by the Ligation Sequencing Kit 1D (PM) without DNA shearing. Afterwards, the size of fragments was detected by an AATI automatic capillary electrophoresis instrument (Agilent Technologies), and the samples were isomolar mixed. Finally, the SQK-LSK109 Connection Kit was used to link the adapter, and the 10K library was constructed. A total amount of 1 µg of the same DNA per sample was used as input material for Illumina sequencing libraries preparation using the NEBNext Ultra DNA Library Prep Kit as per the manufacturer’s protocol. Briefly, DNA was sonicated to 350 bp fragments, then end-polished, A-tailed, ligated with Illumina adapters, and PCR-amplified for purifying and quality checking. The genome of *G. dentisursi* strain 2e was sequenced using the Nanopore PromethION platform (R10.4.1 flow cell) combined with Illumina NovaSeq PE150. Using QC software fastp (v1.3.5), the number of raw Illumina sequencing reads is 5,714,285, and the number of reads after filtering and QC is 4,539,935. We used Dorado basecaller (v7.2.13) with the super-accuracy (sup) model to decode the electrical signals. The number of raw ONT reads is 356,415; the N50 read length is 16,236 bp; and the mean read length is 9,509 bp with a mean read quality of 14.9. We used Unicycler (v0.4.8) (8) with default parameters to assemble and distinguish the chromosomal and plasmid sequences according to sequence length and alignment, and checked the circular genome by whether there is overlap at both ends of contig with trimming the overlapping sequence at the contig ends. PGAP (v6.10) (9) was used to retrieve and annotate the related coding genes by QC of CheckM analysis (v1.2.4) (10).

The complete genome sequence of *G. dentisursi* strain 2e contains a circular chromosome with 5,437,279 bp in length and 55.88% GC content, and two plasmids of 10,765 bp (51.61% GC) and 12,864 bp (56.08% GC) in length. The number of predicted coding genes is 5,293. Subsequently, the genome was rotated to begin with the first base of the *dnaA* gene.

## Data Availability

All sequence data were submitted to GenBank under BioProject ID PRJNA1416607 and Biosample ID SAMN54972261. Raw sequencing data were deposited in the Sequence Read Archive under accession number SRR39406565. The genome sequence has the following accession number: GCA_054956995.1.
